# Metal‐Halide Perovskite Design for Next‐Generation Memories: First‐Principles Screening and Experimental Verification

**DOI:** 10.1002/advs.202001367

**Published:** 2020-06-26

**Authors:** Ju‐Hyun Jung, Seong Hun Kim, Youngjun Park, Donghwa Lee, Jang‐Sik Lee

**Affiliations:** ^1^ Department of Materials Science and Engineering Pohang University of Science and Technology (POSTECH) Pohang 37673 Korea; ^2^ Division of Advanced Materials Science Pohang University of Science and Technology (POSTECH) Pohang 37673 Korea

**Keywords:** CsPb_2_Br_5_, defect formation energy, first‐principle screening, formation energy, resistive switching memory

## Abstract

Memory devices have been advanced so much, but still it is highly required to find stable and reliable materials with low‐power consumption. Halide perovskites (HPs) have been recently adopted for memory application since they have advantages of fast switching based on ionic motion in crystal structure. However, HPs also suffer from poor stability, so it is necessary to improve the stability of HPs. In this regard, combined first‐principles screening and experimental verification are performed to design HPs that have high environmental stability and low‐operating voltage for memory devices. First‐principles screening identifies 2D layered AB_2_X_5_ structure as the best candidate switching layer for memory devices, because it has lower formation energy and defect formation energy than 3D ABX_3_ or other layered structures (A_3_B_2_X_7_, A_2_BX_4_). To verify results, all‐inorganic 2D layered CsPb_2_Br_5_ is synthesized and used in memory devices. The memory devices that use CsPb_2_Br_5_ show much better stability and lower operating voltages than devices that use CsPbBr_3_. These findings are expected to provide new opportunity to design materials for reliable device applications based on calculation, screening, and experimental verification.

## Graphical abstract

A design of halide perovskites (HP) for use in resistive switching memory (RSM) by combining first‐principles screening and experimental verification is conducted. First‐principles calculations identify 2D HP structure (AB_2_X_5_) as the best candidate for RSM. To verify the calculation results, 2D‐layered CsPb_2_Br_5_ is synthesized and applied to RSM. The CsPb_2_Br_5_‐based RSM shows stable operation with low operating voltages.

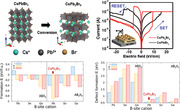

First‐principles density functional theory (DFT) calculations have been used in various fields to determine the physical origin of phenomena, and to predict the electrical properties of materials.^[^ ^1^, ^2^, ^3^, ^4^, ^5^, ^6^, ^7^, ^8^, ^9^, ^10^, ^11^, ^12^, ^13^, ^14^, ^15^, ^16^, ^17^, ^18^ ^]^ DFT calculations have enabled identification of candidate materials by so‐called “high‐throughput screening.”^[^ ^19^, ^20^, ^21^, ^22^ ^]^ For example, DFT calculations have been employed to search for a lead‐free halide perovskites (HPs) photo‐absorber to overcome the presence of toxic lead and thermal instability.^[^ ^23^, ^24^, ^25^ ^]^ In the field of halide HP‐based resistive switching memory (RSM), the use of first‐principles DFT calculations has mainly limited to identifying the origin of electrical characteristics such as low operating voltage, resistive switching failure, and forming‐free behavior.^[^ ^26^, ^27^, ^28^ ^]^ HP‐based RSM has limited stability, so progress in this field requires new materials to improve the stability of HP‐based RSM using first‐principles calculations.

Two strategies have been reported to increase the stability of HP‐based RSM. One strategy is the introduction of a passivation layer such as zinc oxide or polymethyl methacrylate. The passivation layers isolate the HPs from ambient humidity.^[^ ^29^, ^30^, ^31^ ^]^ However, this method induces the problems such as the device complexity and cost of fabrication. Other strategy is to use a low‐dimensional‐layered HP. 2D HP is more stable than a 3D ABX_3_ HP,^[^ ^32^ ^]^ thus use of 2D HP may yield stable RSM devices. However, the experimental approach to find appropriate HPs for use in RSM requires enormous time and effort. Instead, a combination of candidate screening by first‐principles calculation with experimental validation may be an effective method to find candidate HPs for use in RSM. In this study, we combined first‐principles screening and experimental verification to design HPs for use in RSM. We focused on inorganic 2D HPs, which showed better thermal stability than 3D HP structures.^[^ ^33^ ^]^ By comparing formation energy of several 2D perovskite structures, we selected AB_2_X_5_ structure, which has the lowest formation energy, as a candidate structure for use in RSM. To estimate the potential of HP as an RSM material, we used two descriptors: formation energy (*E*_form_) and defect formation energy (*DFE*). Our DFT calculations predicted that CsPb_2_Br_5_ is a good candidate material for RSM because this HP is expected to show better stability with low operating voltage than other inorganic HPs. To verify our calculation results, we fabricated RSM that used CsPb_2_Br_5_ (CsPb_2_Br_5_‐RSM) and compared its resistive switching behavior with RSM that used CsPbBr_3_ (CsPbBr_3_‐RSM). The DFT calculation results matched experimental observations that the CsPb_2_Br_5_‐RSM tended to have lower set electric field than the CsPbBr_3_‐RSM. Also, the CsPb_2_Br_5_‐RSM exhibited resistive switching behavior at 140 °C. Therefore, this combination of first‐principles calculations and experiments identified that CsPb_2_Br_5_ is an appropriate material for use in RSM.

The instability of *α*‐CsPbI_3_ perovskite at room temperature (RT) is a well‐known problem.^[^ ^34^, ^35^, ^36^ ^]^ Also, it is reported that resistive switching property of *α*‐CsPbI_3_‐based RSM was degraded within a short period of time.^[^ ^29^ ^]^ To overcome this problem, we focused on the 2D‐layered HP materials, which are generally known to be more stable than 3D perovskite.^[^ ^33^ ^]^ We considered three 2D‐layered perovskite structures (A_3_B_2_X_7_, A_2_BX_4_, and AB_2_X_5_) to compare their energetic stability with 3D cubic perovskite structure (ABX_3_). We calculated *E*_form_ of four corresponding Cs‐Pb‐I compounds to find most energetically stable structure for inorganic HP (**Figure** **1**a). Our DFT calculation predicted that the *E*_form_ of CsPb_2_I_5_ is the lowest among four different structures (Figure 1b). In addition, there are known techniques stabilizing metastable AB_2_X_5_ structure.^[^ ^37^, ^38^, ^39^ ^]^ Thus, AB_2_X_5_ was chosen as the backbone structure to search for new candidate materials for use in RSM.

**Figure 1:**
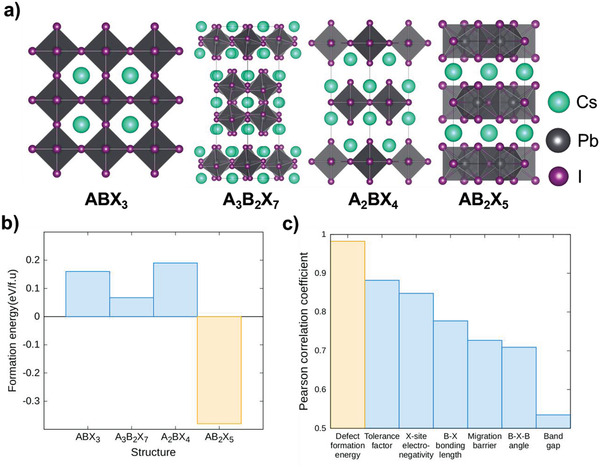
a) Schematics of 3D perovskite (ABX_3_) and 2D‐layered perovskite (A_3_B_2_X_7_, A_2_BX_4_, AB_2_X_5_) structures. b) Comparison on formation energies of ABX_3_, A_3_B_2_X_7_, A_2_BX_4_ and AB_2_X_5_ compounds (A=Cs, B=Pb, X=I). AB_2_X_5_ compound (yellow) shows the lowest formation energy. c) Calculated Pearson correlation coefficients of various materials properties. Defect formation energy (yellow) has the highest correlation with the set voltage.

To find an appropriate composition of AB_2_X_5_ HPs for use in RSM, we first selected two materials properties (*E*_form_ and *DFE*) as descriptors for screening. First, *E*_form_ (eV f.u^−1^., where f.u. is formula unit) was chosen because it can determine the stability of a material.^[^ ^19^, ^40^ ^]^ *E*_form_ of a AB_2_X_5_ compound can be calculated as

(1) $$\begin{array}{c} E_{\mathrm{form}}=E_{\mathrm{tot}}^{\mathrm{bulk}}\;-\left(aE_{\mathrm{tot}}^{\mathrm{AX}}+bE_{\mathrm{tot}}^{BX_{2}}\right) \end{array}$$

where $E_{\mathrm{tot}}^{\mathrm{bulk}}$ is the total energy of the AB_2_X_5_ compound, and $E_{\mathrm{tot}}^{\mathrm{AX}}$ and $E_{\mathrm{tot}}^{BX_{2}}$ are the total energies of the precursors. AX and BX_2_ were chosen as the precursors, because they are widely used precursors in synthesis of layered HP.^[^ ^41^, ^42^ ^]^ The stoichiometry of the AB_2_X_5_ compound was maintained by choosing *a* and *b* (*a* = 1, *b* = 2) appropriately.

Second, *DFE* of halide vacancy $\left(V_{X}^{•}\right)$ was chosen since the Pearson correlation analysis predicts that it is the most correlated materials property with the operating (set) voltage (*V*_OP_) of an RSM device. In this study, candidate materials that can minimize the energy consumption of RSM by reducing *V*_OP_ are investigated. To identify material characteristics that are most correlated with *V*_OP_, we calculated Pearson correlation coefficients with various materials properties such as *DFE* of $V_{X}^{•},$ tolerance factor, electronegativity of X‐site halide ion, average bonding length between B‐site cation and X‐site anion, migration barrier of a halide ion, in‐plane bonding angle between two B—X bonds, and band gap (Figure 1c). Detail descriptions on the Pearson correlation analysis are provided in Table S1, Supporting Information. Our analysis predicted that the *DFE* of $V_{X}^{•}$ was the most strongly correlated with *V*_OP_. $V_{X}^{•}$ participates in the formation and rupture of conducting filaments for resistance switching,^[^ ^26^, ^43^, ^44^, ^45^, ^46^ ^]^ so the high correlation is quite reasonable. The *DFE* (eV) of $V_{X}^{•}$ ($\mathrm{DFE}[V_{X}^{•}]$) can be calculated as

(2) $$\begin{array}{c} \mathrm{DFE}\left[V_{X}^{•}]\;=E_{\mathrm{tot}}\;[V_{X}^{•}\right]-E_{\mathrm{tot}}^{\mathrm{bulk}}+\mu_{x}+q\mu_{e} \end{array}$$

where $E_{\mathrm{tot}}\left[V_{X}^{•}\right]$ is the total energy of the defective system with $V_{X}^{•},$ $E_{\mathrm{tot}}^{\mathrm{bulk}}$ is the total energy of a perfect system, *μ*_*x*_ is the chemical potential of an X anion, *q* is the charge of a vacancy, and *μ*_*e*_ is the chemical potential of an electron; here, the valence‐band maximum of the perfect system is used for *μ*_*e*_. First‐principles DFT calculations were employed to calculate *E*_form_ and *DFE* of $V_{X}^{•}$ to find candidate materials that have AB_2_X_5_ structure for use in RSM. The A‐site was fixed as Cs^+^, the B‐site was a divalent cation (Pb^2+^, Sn^2+^, Ge^2+^), and the X‐site was a halide anion (Cl^−^, Br^−^, I^−^). To compare the properties of AB_2_X_5_ structure, the properties of ABX_3_ structure were also calculated. Our DFT calculations predicted that *E*_form_ of AB_2_X_5_ structure is lower than that of ABX_3_ structure when Pb^2+^ and Sn^2+^ sit on B‐site (**Figure** **2**a). Especially, CsPb_2_Br_5_ (−0.88 eV f.u.^−1^) and CsPb_2_Cl_5_ (−0.83 eV f.u.^−1^) are the two lowest *E*_form_ compounds. The results on the *DFE* of $V_{X}^{•}$ show that halide vacancies can be preferentially formed in the order of I, Br, and Cl (Figure 2b). Since the bonding strength between B‐site and X‐site ions due to the electronegativity difference increases as ascending the halide column in the periodic table, the observed trend is reasonable. In addition, the *DFE* of $V_{X}^{•}$ is always lower in AB_2_X_5_ than that in ABX_3_ of same composition. Especially, CsGe_2_I_5_ (0.53 eV), CsPb_2_I_5_ (0.53 eV), CsGe_2_Br_5_ (0.61 eV), and CsPb_2_Br_5_ (0.61 eV) show relatively low *DFE* of $V_{X}^{•}$. Thus, they are expected to show high $V_{X}^{•}$ concentration and therefore a low set electric field for resistive switching.

**Figure 2:**
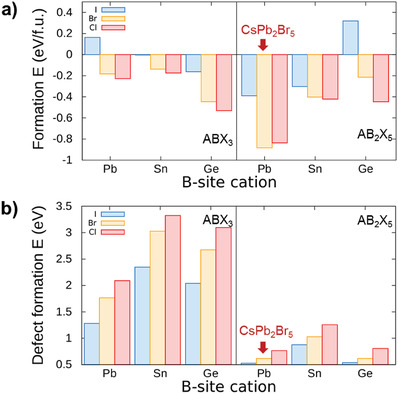
a) Formation energy of CsBX_3_ (left) and CsB_2_X_5_ (right). b) Defect formation energy of a halide vacancy in CsBX_3_ (left) and CsB_2_X_5_ (right). The color of the bars indicates the type of X‐site halide (blue: I, orange: Br, red: Cl). Red arrow indicates most adequate material for resistive switching memory.

The most suitable candidate for use in RSM is chosen by comparing *E*_form_ and *DFE* of various compounds. The *E*_form_ and *DFE* seem to be inversely proportional to each other, but the DPE defines how much the defective system can be stabilized compared to the perfect system. Therefore, the system can have low DFE even if *E*_form_ is low from our calculation results. As can be seen from Figure 2, most of ABX_3_ compounds are not suitable materials for RSM due to their high *E*_form_ and *DFE* of $V_{X}^{•}$. On the other hand, many AB_2_X_5_ compounds show relative lower *E*_form_ and *DFE* of $V_{X}^{•}$. Especially, CsPb_2_Br_5_ had the lowest *E*_form_ and quite low *DFE* of $V_{X}^{•}$. Although CsGe_2_I_5_ and CsGe_2_Br_5_ had relatively low *DFE* of $V_{X}^{•}$, they had high *E*_form_, so they were not suitable materials for RSM. In contrast, CsPb_2_Cl_5_ had low *E*_form_, but high *DFE* of $V_{X}^{•}$, so it was not suitable as well. In this study, thus CsPb_2_Br_5_ was selected as the active layer of RSM to ensure the maximum stability and low *V*_OP_.

Among various Cs‐Pb‐Br compounds, the orthorhombic‐CsPbBr_3_ has already been reported as an RSM active material^[^ ^47^, ^48^ ^]^ and known to be stable phase in CsPbBr_3_ composition at RT.^[^ ^49^ ^]^ The screening calculations in the previous section considered cubic‐ABX_3_ structure, so we further calculated the *E*_form_ and the *DFE* of $V_{\mathrm{Br}}^{•}$ of orthorhombic‐CsPbBr_3_ and compared them with those of CsPb_2_Br_5_ to verify its feasibility as an RSM active material. Our DFT calculations predicted that *E*_form_ of CsPb_2_Br_5_ (−0.88 eV f.u.^−1^) is much lower than that of orthorhombic‐CsPbBr_3_ (−0.45 eV f.u.^−1^), thus CsPb_2_Br_5_ is more stable than orthorhombic‐CsPbBr_3_. Also, the calculated *DFE* of $V_{\mathrm{Br}}^{•}$ for CsPb_2_Br_5_ (0.61 eV) is lower than that for orthorhombic‐CsPbBr_3_ (1.24 eV). So, we confirm that CsPb_2_Br_5_ can have better stability and lower set electric field than orthorhombic‐CsPbBr_3_. To conclude, our DFT study predicts that CsPb_2_Br_5_ has superior characteristics for use in RSM.

We synthesized CsPb_2_Br_5_ and CsPbBr_3_ as resistive switching layers to confirm the calculation results and to compare the device performance such as set electric field and ON/OFF ratio. Water vapor–induced transformation was used to transform CsPbBr_3_ to CsPb_2_Br_5_. A CsPbBr_3_ precursor was spin‐coated on the indium tin oxide (ITO)–coated glass substrate (**Figure** **3**a). The deposited film was annealed at 100 °C to remove the residual solvent. To form a CsPb_2_Br_5_ film, the deposited CsPbBr_3_ film was converted to CsPb_2_Br_5_ by using a home‐made humidity chamber (85% relative humidity (RH), RT). CsPb_2_Br_5_ has a 2D structure, which is composed of Cs^+^ cations sandwiched between [Pb_2_Br_5_]^−^ layers (Figure 3b); this structure is obtained from 3D CsPbBr_3_, which has a cubic structure with corner‐sharing [PbBr_6_]^4−^ octahedral units as^[^ ^50^ ^]^

(3) $$\begin{array}{c} 2\mathrm{CsPbB}r_{3}\to \mathrm{CsP}b_{2}Br_{5}+Cs^{+}\left(\mathrm{aq}\right)+Br^{-}\left(\mathrm{aq}\right) \end{array}$$

**Figure 3:**
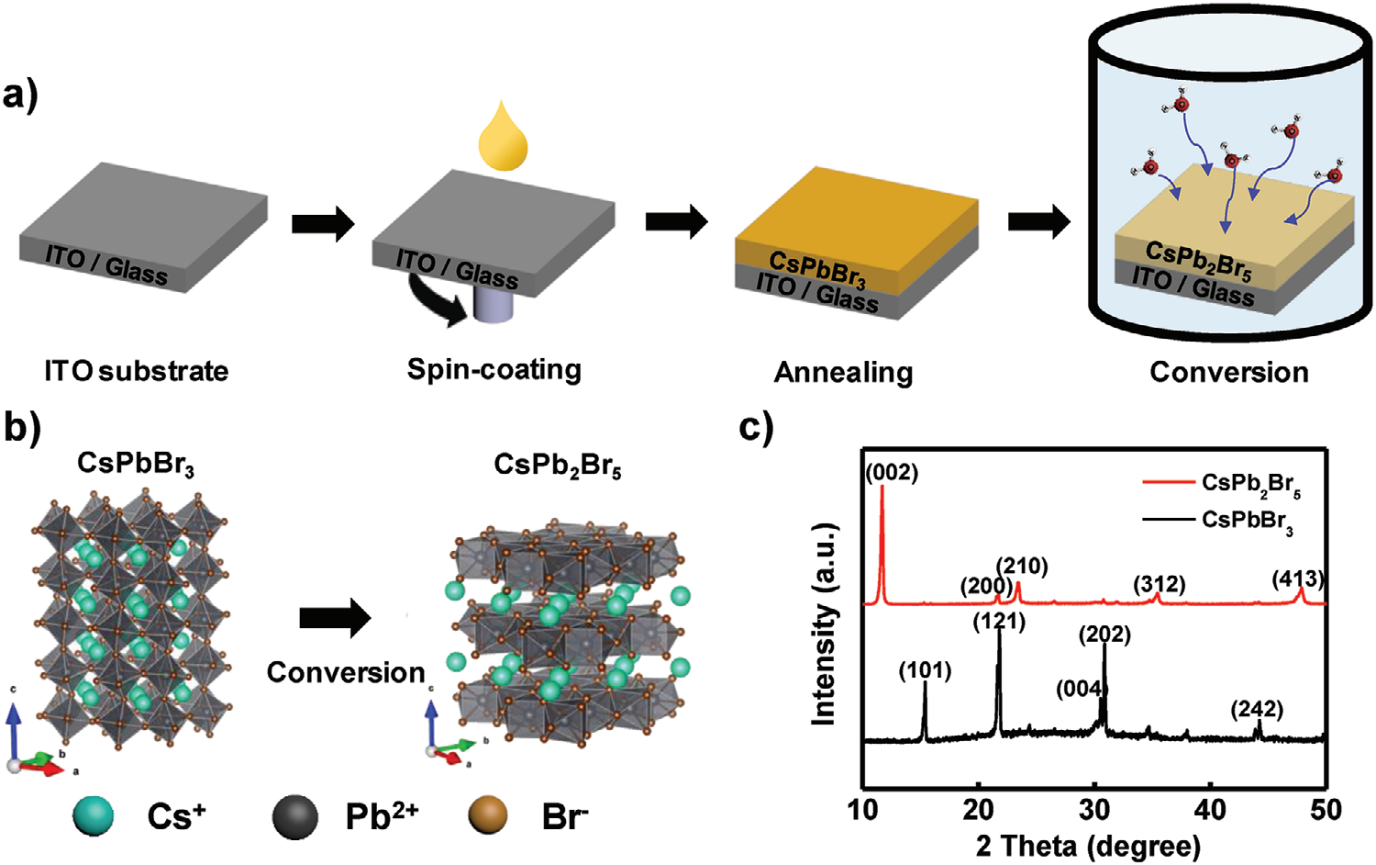
a) Schematic experimental procedure to synthesize CsPbBr_3_ using solution process and CsPb_2_Br_5_ using water‐assisted transformation. b) Phase transformation from CsPbBr_3_ to CsPb_2_Br_5_. c) XRD patterns of CsPbBr_3_ and CsPb_2_Br_5_.

When the CsPbBr_3_ reacts with water molecules during water treatment, Cs^+^ and Br^−^ are extracted from the CsPbBr_3_, so it decomposes to PbBr_2_, Cs^+^(aq), and Br^−^(aq). This change occurs because CsPbBr_3_ has ionic nature, and the cesium bromide (CsBr) has high solubility in water. If lead bromide (PbBr_2_) is abundant, it crystalizes with CsBr to form tetragonal CsPb_2_Br_5_, which is thermodynamically stable in humid air.^[^ ^50^ ^]^

The X‐ray diffraction (XRD) patterns of CsPbBr_3_ and transformed CsPb_2_Br_5_ films were investigated at incidence angles 10° ≤ 2*θ* ≤ 50°. The XRD patterns (Figure 3c, black line) of CsPbBr_3_ indicated an orthorhombic phase that exhibited peaks at 15.4°, 21.8°, 30.5°, 30.9° and 44.2°, which could be assigned to (101), (121), (004), (202), and (242) planes, respectively. The XRD patterns of CsPb_2_Br_5_ (Figure 3c, red line) were tetragonal phase that showed peaks at 11.7°, 21.6°, 23.4°, 35.4°, and 47.9°, which could be indexed to (002), (200), (210), (312), and (413) planes, respectively; the CsPb_2_Br_5_ film did not show peaks that indicated the presence of PbBr_2_ or CsPbBr_3_. These results showed that the water‐vapor treatment induced complete phase transformation from orthorhombic CsPbBr_3_ to tetragonal CsPb_2_Br_5_.^[^ ^42^ ^]^ The CsPbBr_3_ and CsPb_2_Br_5_ deposited on the ITO‐coated glass substrate showed uniform perovskite layers with thickness *t*_P_ = 406  and 523 nm, respectively (Figure S1, Supporting Information).

The current‐voltage (*I*–*V*) characteristics of Au/CsPbBr_3_/ITO and Au/CsPb_2_Br_5_/ITO devices were measured under ambient air. During these electrical characterizations, DC bias voltages (*V*) were applied to the Au top electrodes in the sequence of 0  → 1.5  → 0  → −1.5  → 0 V, while the ITO (bottom electrode) was grounded. These devices showed bipolar resistive switching behavior under a compliance current (CC) = 10^−4^ A (**Figure** **4**a). In this work, we used electric field (*E = V/t*_P_) instead *V* to compensate for the difference in *t*_P_ of the CsPbBr_3_ and CsPb_2_Br_5_ films. The set process occurred at 13.7 kV cm^−1^ for CsPbBr_3_, and 10.4 kV cm^−1^ for CsPb_2_Br_5_; each was the average taken from 10 individual devices (Figure S2, Supporting Information). The set electric field (*E*_SET_) of CsPb_2_Br_5_‐RSM was 1.32 times lower than the CsPbBr_3_‐RSM (Figure 4b). This difference confirmed the DFT calculation results that *E*_SET_ should be lower in 2D CsPb_2_Br_5_ film than in 3D CsPbBr_3_ film. The difference may occur because DFE of $V_{\mathrm{Br}}^{•}$ is lower in CsPb_2_Br_5_ than in CsPbBr_3_, so conductive filaments may be easily formed in CsPb_2_Br_5_.

**Figure 4:**
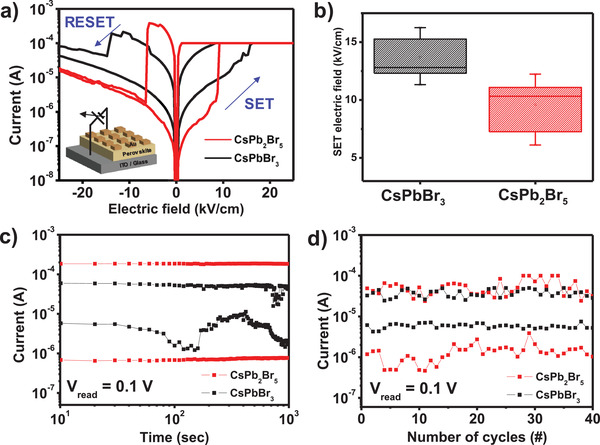
Programmable memory properties of Au/CsPbBr_3_/ITO and Au/CsPb_2_Br_5_/ITO devices. a) *I*–*V* characteristics of the Au/CsPbBr_3_/ITO and Au/CsPb_2_Br_5_/ITO. b) The statistical distribution of set electric field. c) Data retention properties of Au/CsPbBr_3_/ITO and Au/CsPb_2_Br_5_/ITO. d) Endurance properties of Au/CsPbBr_3_/ITO and Au/CsPb_2_Br_5_/ITO.

Data retention and endurance were measured to confirm the reliability of the memory device with a reading voltage of 0.1 V (Figure 4c,d). The CsPbBr_3_‐RSM and CsPb_2_Br_5_‐RSM showed a clear difference between a low‐resistance state (LRS) and high‐resistance state (HRS) for at least 10^3^ s, and the electrical properties of the devices showed almost no change for 40 cycles. Also, we confirmed the endurance properties using pulse measurement. Set voltage pulses (2 V, 50 ms) and reset voltage pulses (−1 V, 50 ms) were applied to induce LRS and HRS, respectively. Each resistance state was measured at read voltage (0.1 V, 100 ms) under CC = 10^−4^ A. The device showed distinguishable LRS and HRS over 500 cycles without degradation (Figure S3, Supporting Information). The ON/OFF ratio of the CsPb_2_Br_5_‐RSM was higher than that of the CsPbBr_3_‐RSM. The difference was a result of a decreased HRS current in CsPb_2_Br_5_. The reduced dimensionality of CsPb_2_Br_5_ increases the Schottky barrier height between the electrode and CsPb_2_Br_5_, and this can lead to high ON/OFF ratio of 2D perovskite‐based RSM.^[^ ^44^ ^]^ A comparison between previous HP‐based RSM and our devices was made (Table S2, Supporting Information).^[^ ^47^, ^51^, ^52^, ^53^ ^]^ CsPb_2_Br_5_‐RSM in this study showed memory characteristics comparable to previously reported HP‐based RSM devices.

To understand the conduction mechanism, we replotted the *I–V* curves of CsPbBr_3_‐ and CsPb_2_Br_5_‐RSM on a full‐logarithmic scale (Figure S4, Supporting Information). In the LRS state, the fitted curves showed slopes near 1 (*I*∝*V* ^1.03^ for CsPbBr_3_, *I*∝*V* ^0.97^ for CsPb_2_Br_5_), which indicated that both devices showed Ohmic conduction behavior. This result showed that the conductive filaments might be formed between the top electrode (Au) and the bottom electrode (ITO).^[^ ^51^ ^]^ In the HRS state, the conduction mechanism consisted of two parts. At low voltage, the curves showed Ohmic conduction behavior (*I*∝*V* ^1.09^ for CsPbBr_3_, *I*∝*V* ^1.07^ for CsPb_2_Br_5_) as in the LRS state. However, at high voltage region, the fitted curves almost conformed to Child's law (*I*∝*V* ^2.33^ for CsPbBr_3_, *I*∝*V* ^2.32^ for CsPb_2_Br_5_), which indicated that the mechanism had changed to trap‐filled space‐charge‐limited conduction.

The poor stability of HPs impedes its use in practical memory devices. DFT calculations suggested that CsPb_2_Br_5_ has low *E*_form_ (−0.88 eV f.u.^−1^), so it is expected to be more thermodynamically stable than CsPbBr_3_. To evaluate the thermal stability of CsPb_2_Br_5_‐ and CsPbBr_3_‐RSM, the *I–V* characteristics and XRD patterns of CsPb_2_Br_5_ and CsPbBr_3_ were measured at elevated temperatures (**Figure** **5**). The CsPb_2_Br_5_‐RSM showed a bipolar resistive switching behavior even after being heated to 140 °C (Figure 5a). We measured the data retention at 60, 100, and 140 °C and the CsPb_2_Br_5_‐RSM showed stable retention characteristics for 10^3^ s (Figure S5, Supporting Information). Also, we confirmed the thermal stability of the states for 10^4^ s at 60 °C (Figure S6, Supporting Information). However, the electrical properties of CsPbBr_3_‐RSM were degraded with a rapid drop in ON/OFF ratio at 140 °C (Figure 5b,c). To check the variation of the films, the XRD patterns of CsPb_2_Br_5_ and CsPbBr_3_ thin films were measured after annealing at 140 °C for 30 min in ambient air. CsPb_2_Br_5_ film did not show change (Figure 5d), but CsPbBr_3_ film showed change in XRD patterns after annealing (Figure S7, Supporting Information). In the annealed CsPbBr_3_ thin film, the additional phases were observed, such as CsPb_2_Br_5_, CsBr, and PbBr_2_. The phase evolution from CsPbBr_3_ to CsPb_2_Br_5_ during annealing in ambient air might be caused by the moisture and the difference in Gibbs free energy of the reaction.^[^ ^54^ ^]^ Also, due to high ionic character of the CsPbBr_3_, the decomposition of CsPbBr_3_ might occur by the moisture and heat.^[^ ^55^ ^]^ This degradation in the annealed CsPbBr_3_ thin film can cause malfunction of CsPbBr_3_‐RSM at 140 °C. These results suggest that the CsPb_2_Br_5_‐RSM is thermally stable in the air.

**Figure 5:**
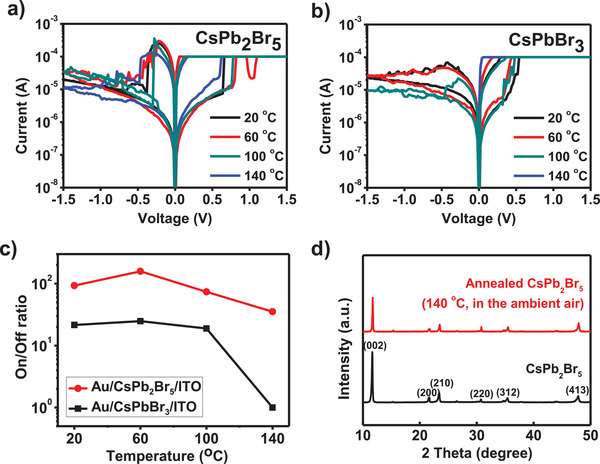
*I–V* characteristics of the a) Au/CsPb_2_Br_5_/ITO device and b) Au/CsPbBr_3_/ITO device measured at 20, 60, 100, and 140 °C in ambient air. c) ON/OFF ratio of Au/CsPb_2_Br_5_/ITO and Au/CsPbBr_3_/ITO devices at 20, 60, 100, and 140 °C. d) XRD patterns before and after annealing of CsPb_2_Br_5_ at 140 °C in ambient air.

In conclusion, first‐principles screening was combined with experimental verification to design new HPs for use in RSM. Two descriptors (*E*_form_ and *DFE* of $V_{X}^{•}$) were chosen to screen candidate materials for RSM because they were closely related to stability and operating voltage. Our DFT calculation predicted that 2D HP structure (AB_2_X_5_) would be more stable and have lower set electric field than 3D HP structure (ABX_3_) due to the low *E*_form_ and *DFE* of $V_{\mathrm{Br}}^{•}$. Among the various composition of AB_2_X_5_ HPs, DFT calculations predicted that CsPb_2_Br_5_ would be the best candidate for use in RSM. To confirm the prediction, we fabricated Au/CsPbBr_3_/ITO and Au/CsPb_2_Br_5_/ITO devices to compare their resistive switching behaviors. The CsPb_2_Br_5_‐RSM had lower set electric field and higher ON/OFF ratio than the CsPbBr_3_‐RSM. CsPb_2_Br_5_‐RSM retained resistive switching behavior even at 140 °C. Therefore, we confirmed that the CsPb_2_Br_5_‐RSM showed a superior resistive switching behavior and thermodynamic stability than CsPbBr_3_‐RSM. These results provide a promising route to design HPs that are suitable for use in RSM.

## Experimental Section

##### Materials

Cesium acetate (CsOAc, ≥ 95% purity), lead bromide (PbBr_2_, ≥ 98% purity), dimethyl sulfoxide (DMSO, ≥ 99.9% purity), dimethylformamide (DMF, ≥ 99.8% purity), and ITO‐coated glass substrates were purchased from Sigma‐Aldrich. All materials were used without further purification.

##### Deposition of Perovskite Films and Device Fabrication

First, 1 m of CsOAc and 2 m of PbBr_2_ were dissolved in DMSO, separately. The prepared solution was stirred on a hot plate at 100 °C. A 0.5 mL of each solution was dissolved in 0.5 mL of DMF together. The solution was filtered using a 0.2‐µm pore‐size filter. Before the spin‐coating process, the ITO/glass substrate was cleaned in ethanol, acetone, deionized water for 10 min each, and treated with UV/O_3_ (wavelength = 253.7 and 184.9 nm) for 20 min. The solution was spin‐coated at 900 rpm for 10 s, then annealed at 100 °C for 10 min to form the CsPbBr_3_ perovskite film. To form CsPb_2_Br_5_ film, the CsPbBr_3_ perovskite film was held in a home‐made humidity chamber with 85% RH until the CsPbBr_3_ had completely converted to CsPb_2_Br_5_. Au electrodes were deposited on the HP films by thermal evaporation through a shadow mask.

##### Characterization

The crystal structure of CsPbBr_3_ and CsPb_2_Br_5_ was measured using XRD (Rigaku D/MAX‐2500 Diffractometer) with Cu K*α* radiation. Current‐voltage characteristics were measured using a semiconductor parameter analyzer (4200a‐SCS, KEITHLEY) in the probe station in ambient condition. The microstructure as observed using high‐resolution field‐emission SEM (JSM 7800, JEOL).

##### Calculation Section—Methodology

First‐principles DFT calculations were used to identify candidate perovskites for use as an active material for RSM and, to understand the physical origin of resistive switching in RSM based on CsPb_2_Br_5_. The DFT calculations were conducted using the projector augmented wave ^[^ ^56^ ^]^ method with the Perdew–Burke–Ernzerhof functional for exchange and correlation potentials,^[^ ^57^, ^58^ ^]^ as implemented in the Vienna Ab‐initio Simulation Package code.^[^ ^59^, ^60^ ^]^ For calculations of various AB_2_X_5_ and ABX_3_ compounds, structures with 8 formula unit (8 f.u.) were constructed and Monkhorst‐Pack k‐point^[^ ^61^ sampling with 4 × 4 × 4 grids was used. For further calculations for CsPb_2_Br_5_ and orthorhombic‐CsPbBr_3_, 16 f.u. structures with a 2 × 2 × 2 k‐point grid were used. To consider the interlayer interactions the van der Waals correction by using DFT‐D3^[^ ^62^ ^]^ method was used. The total energy was converged to within 1 meV with a kinetic energy cut‐off of 500 eV for all calculations.

## Conflict of Interest

The authors declare no conflict of interest.

## Author Contributions

J.‐S.L. conceived and directed the research. D.L. directed the calculation part. J.H.J. and Y.P. performed the experiment and acquired the data. S.H.K. and D.L. performed the first‐principle calculations. Y.P., S.H.K., D.L., and J.‐S.L. revised the manuscript. J.H.J., S.H.K. and Y.P. contributed equally to this work.

## Supporting information

Supporting Information

## References

[1] J. A. Hachtel , J. Huang , I. Popovs , S. Jansone‐Popova , J. K. Keum , J. Jakowski , T. C. Lovejoy , N. Dellby , O. L. Krivanek , J. C. Idrobo , Science 2019, 363, 525. 30705191 10.1126/science.aav5845

[2] A.‐K. Henß , S. Sakong , P. K. Messer , J. Wiechers , R. Schuster , D. C. Lamb , A. Groß , J. Wintterlin , Science 2019, 363, 715. 30765561 10.1126/science.aav4143

[3] T. Zhang , Y. Jiang , Z. Song , H. Huang , Y. He , Z. Fang , H. Weng , C. Fang , Nature. 2019, 566, 475. 30814713 10.1038/s41586-019-0944-6

[4] A. Govind Rajan , K. S. Silmore , J. Swett , A. W. Robertson , J. H. Warner , D. Blankschtein , M. S. Strano , Nat. Mater. 2019, 18, 129. 30643239 10.1038/s41563-018-0258-3

[5] C. Motta , F. El‐Mellouhi , S. Kais , N. Tabet , F. Alharbi , S. Sanvito , Nat. Commun. 2015, 6, 7026. 25912782 10.1038/ncomms8026 PMC4421841

[6] J. Even , L. Pedesseau , J.‐M. Jancu , C. Katan , Phys. Status Solidi Rapid Res. Lett. 2014, 8, 31.

[7] J. Y. Raty , W. Zhang , J. Luckas , C. Chen , R. Mazzarello , C. Bichara , M. Wuttig , Nat. Commun. 2015, 6, 7467. 26105012 10.1038/ncomms8467

[8] D. H. Kwon , S. Lee , C. S. Kang , Y. S. Choi , S. J. Kang , H. L. Cho , W. Sohn , J. Jo , S. Y. Lee , K. H. Oh , T. W. Noh , R. A. De Souza , M. Martin , M. Kim , Adv. Mater. 2019, 31, 1901322. 10.1002/adma.201901322 31106484

[9] W. Ding , J. Zhu , Z. Wang , Y. Gao , D. Xiao , Y. Gu , Z. Zhang , W. Zhu , Nat. Commun. 2017, 8, 14956. 28387225 10.1038/ncomms14956 PMC5385629

[10] P. C. Harikesh , B. Wu , B. Ghosh , R. A. John , S. Lie , K. Thirumal , L. H. Wong , T. C. Sum , S. Mhaisalkar , N. Mathews , Adv. Mater. 2018, 30, 1802080. 10.1002/adma.201802080 29978516

[11] B. Anasori , M. R. Lukatskaya , Y. Gogotsi , Nat. Rev. Mater. 2017, 2, 16098.

[12] J. Besnardiere , B. Ma , A. Torres‐Pardo , G. Wallez , H. Kabbour , J. M. Gonzalez‐Calbet , H. J. Von Bardeleben , B. Fleury , V. Buissette , C. Sanchez , T. Le Mercier , S. Cassaignon , D. Portehault , Nat. Commun. 2019, 10, 327. 30659185 10.1038/s41467-018-07774-x PMC6338762

[13] H. Tsai , W. Nie , J.‐C. Blancon , C. C. Stoumpos , R. Asadpour , B. Harutyunyan , A. J. Neukirch , R. Verduzco , J. J. Crochet , S. Tretiak , L. Pedesseau , J. Even , M. A. Alam , G. Gupta , J. Lou , P. M. Ajayan , M. J. Bedzyk , M. G. Kanatzidis , A. D. Mohite , Nature. 2016, 536, 312. 27383783 10.1038/nature18306

[14] J. A. Mundy , C. M. Brooks , M. E. Holtz , J. A. Moyer , H. Das , A. F. Rébola , J. T. Heron , J. D. Clarkson , S. M. Disseler , Z. Liu , A. Farhan , R. Held , R. Hovden , E. Padgett , Q. Mao , H. Paik , R. Misra , L. F. Kourkoutis , E. Arenholz , A. Scholl , J. A. Borchers , W. D. Ratcliff , R. Ramesh , C. J. Fennie , P. Schiffer , D. A. Muller , D. G. Schlom , Nature. 2016, 537, 523. 27652564 10.1038/nature19343

[15] P. Umari , E. Mosconi , F. De Angelis , Sci. Rep. 2015, 4, 4467. 10.1038/srep04467 PMC5394751 24667758

[16] C. Wolf , T.‐W. Lee , Mater. Today Energy 2018, 7, 199.

[17] F. Brivio , C. Caetano , A. Walsh , J. Phys. Chem. Lett. 2016, 7, 1083. 26952337 10.1021/acs.jpclett.6b00226 PMC5042358

[18] S. H. Kim , D. Lee , J. Phys. Chem. C 2019, 123, 9629.

[19] S. Curtarolo , G. L. Hart , M. B. Nardelli , N. Mingo , S. Sanvito , O. Levy , Nat. Mater. 2013, 12, 191. 23422720 10.1038/nmat3568

[20] A. R. Oganov , C. J. Pickard , Q. Zhu , R. J. Needs , Nat. Rev. Mater. 2019, 4, 331.

[21] O. Senkov , J. Miller , D. Miracle , C. Woodward , Nat. Commun. 2015, 6, 6529. 25739749 10.1038/ncomms7529 PMC4366518

[22] N. Mounet , M. Gibertini , P. Schwaller , D. Campi , A. Merkys , A. Marrazzo , T. Sohier , I. E. Castelli , A. Cepellotti , G. Pizzi , Nat. Nanotechnol. 2018, 13, 246. 29410499 10.1038/s41565-017-0035-5

[23] T. Nakajima , K. Sawada , J. Phys. Chem. Lett. 2017, 8, 4826. 28927268 10.1021/acs.jpclett.7b02203

[24] X.‐G. Zhao , D. Yang , Y. Sun , T. Li , L. Zhang , L. Yu , A. Zunger , J. Am. Chem. Soc. 2017, 139, 6718. 28430435 10.1021/jacs.7b02120

[25] X.‐G. Zhao , J.‐H. Yang , Y. Fu , D. Yang , Q. Xu , L. Yu , S.‐H. Wei , L. Zhang , J. Am. Chem. Soc. 2017, 139, 2630. 28112933 10.1021/jacs.6b09645

[26] B. Hwang , C. Gu , D. Lee , J.‐S. Lee , Sci. Rep. 2017, 7, 43794. 28272547 10.1038/srep43794 PMC5341555

[27] J.‐M. Yang , S.‐G. Kim , J.‐Y. Seo , C. Cuhadar , D.‐Y. Son , D. Lee , N.‐G. Park , Adv. Electron. Mater. 2018, 4, 1800190.

[28] C. Cuhadar , S. G. Kim , J. M. Yang , J. Y. Seo , D. Lee , N. G. Park , ACS Appl. Mater. Interfaces 2018, 10, 29741. 29968458 10.1021/acsami.8b07103

[29] J. S. Han , Q. V. Le , J. Choi , K. Hong , C. W. Moon , T. L. Kim , H. Kim , S. Y. Kim , H. W. Jang , Adv. Funct. Mater. 2018, 28, 1705783.

[30] B. Hwang , J.‐S. Lee , Sci. Rep. 2017, 7, 673. 28386084 10.1038/s41598-017-00778-5 PMC5429663

[31] Y. Wang , Z. Lv , Q. Liao , H. Shan , J. Chen , Y. Zhou , L. Zhou , X. Chen , V. A. L. Roy , Z. Wang , Z. Xu , Y. J. Zeng , S. T. Han , Adv. Mater. 2018, 30, 1800327. 10.1002/adma.201800327 29782667

[32] L. N. Quan , M. Yuan , R. Comin , O. Voznyy , E. M. Beauregard , S. Hoogland , A. Buin , A. R. Kirmani , K. Zhao , A. Amassian , D. H. Kim , E. H. Sargent , J. Am. Chem. Soc. 2016, 138, 2649. 26841130 10.1021/jacs.5b11740

[33] G. Grancini , M. K. Nazeeruddin , Nat. Rev. Mater. 2019, 4, 4.

[34] A. Marronnier , H. Lee , B. Geffroy , J. Even , Y. Bonnassieux , G. Roma , J. Phys. Chem. Lett. 2017, 8, 2659. 28553717 10.1021/acs.jpclett.7b00807

[35] T. Zhang , M. I. Dar , G. Li , F. Xu , N. Guo , M. Grätzel , Y. Zhao , Sci. Adv. 2017, 3, e1700841. 28975149 10.1126/sciadv.1700841 PMC5621977

[36] L. Meng , J. You , Y. Yang , Nat. Commun. 2018, 9, 5265. 30532038 10.1038/s41467-018-07255-1 PMC6288125

[37] S. K. Balakrishnan , P. V. Kamat , Chem. Mater. 2018, 30, 74.

[38] Q. A. Akkerman , S. Park , E. Radicchi , F. Nunzi , E. Mosconi , F. De Angelis , R. Brescia , P. Rastogi , M. Prato , L. Manna , Nano Lett. 2017, 17, 1924. 28196323 10.1021/acs.nanolett.6b05262 PMC5345893

[39] L. Wu , H. Hu , Y. Xu , S. Jiang , M. Chen , Q. Zhong , D. Yang , Q. Liu , Y. Zhao , B. Sun , Nano Lett. 2017, 17, 5799. 28806517 10.1021/acs.nanolett.7b02896

[40] I. E. Castelli , T. Olsen , S. Datta , D. D. Landis , S. Dahl , K. S. Thygesen , K. W. Jacobsen , Energy Environ. Sci. 2012, 5, 5814.

[41] K. H. Wang , L. Wu , L. Li , H. B. Yao , H. S. Qian , S. H. Yu , Angew. Chem., Int. Ed. 2016, 55, 8328. 10.1002/anie.201602787 27213688

[42] M. Liu , J. Zhao , Z. Luo , Z. Sun , N. Pan , H. Ding , X. Wang , Chem. Mater. 2018, 30, 5846.

[43] B. Hwang , J.‐S. Lee , Adv. Mater. 2017, 29, 1701048. 10.1002/adma.201701048 28558134

[44] J. Y. Seo , J. Choi , H. S. Kim , J. Kim , J. M. Yang , C. Cuhadar , J. S. Han , S. J. Kim , D. Lee , H. W. Jang , N. G. Park , Nanoscale 2017, 9, 15278. 28994433 10.1039/c7nr05582j

[45] Y. Sun , M. Tai , C. Song , Z. Wang , J. Yin , F. Li , H. Wu , F. Zeng , H. Lin , F. Pan , J. Phys. Chem. C 2018, 122, 6431.

[46] E. J. Yoo , M. Lyu , J. H. Yun , C. J. Kang , Y. J. Choi , L. Wang , Adv. Mater. 2015, 27, 6170. 26331363 10.1002/adma.201502889

[47] D. Liu , Q. Lin , Z. Zang , M. Wang , P. Wangyang , X. Tang , M. Zhou , W. Hu , ACS Appl. Mater. Interfaces 2017, 9, 6171. 28112895 10.1021/acsami.6b15149

[48] Q. Lin , W. Hu , Z. Zang , M. Zhou , J. Du , M. Wang , S. Han , X. Tang , Adv. Electron. Mater. 2018, 4, 1700596.

[49] Y. He , L. Matei , H. J. Jung , K. M. McCall , M. Chen , C. C. Stoumpos , Z. Liu , J. A. Peters , D. Y. Chung , B. W. Wessels , Nat. Commun. 2018, 9, 1609. 29686385 10.1038/s41467-018-04073-3 PMC5913317

[50] B. Turedi , K. J. Lee , I. Dursun , B. Alamer , Z. Wu , E. Alarousu , O. F. Mohammed , N. Cho , O. M. Bakr , J. Phys. Chem. C 2018, 122, 14128.

[51] C. Gu , J.‐S. Lee , ACS Nano 2016, 10, 5413. 27093096 10.1021/acsnano.6b01643

[52] E. J. Yoo , M. Lyu , J.‐H. Yun , C. J. Kang , Y. J. Choi , L. Wang , Adv. Mater. 2015, 27, 6170. 26331363 10.1002/adma.201502889

[53] R. Chen , J. Xu , M. Lao , Z. Liang , Y. Chen , C. Zhong , L. Huang , A. Hao , M. Ismail , Phys. Status Solidi RRL 2019, 13, 1900397.

[54] G. Li , H. Wang , Z. Zhu , Y. Chang , T. Zhang , Z. Song , Y. Jiang , Chem. Commun. 2016, 52, 11296. 10.1039/c6cc05877a 27709195

[55] B. Akbali , G. Topcu , T. Guner , M. Ozcan , M. M. Demir , H. Sahin , Phys. Rev. Mater. 2018, 2, 034601.

[56] P. E. Blöchl , Phys. Rev. B 1994, 50, 17953. 10.1103/physrevb.50.17953 9976227

[57] J. P. Perdew , K. Burke , M. Ernzerhof , Phys. Rev. Lett. 1996, 77, 3865. 10062328 10.1103/PhysRevLett.77.3865

[58] J. P. Perdew , K. Burke , Y. Wang , Phys. Rev. B 1998, 57, 14999.

[59] M. Fuchs , M. Scheffler , Comput. Phys. Commun. 1999, 119, 67.

[60] G. Kresse , J. Furthmüller , Phys. Rev. B 1996, 54, 11169. 10.1103/physrevb.54.11169 9984901

[61] H. J. Monkhorst , J. D. Pack , Phys. Rev. B 1976, 13, 5188.

[62] S. Grimme , J. Antony , S. Ehrlich , H. Krieg , J. Chem. Phys. 2010, 132, 154104. 20423165 10.1063/1.3382344

