# Supplementary material for: Metal‐Halide Perovskite Design for Next‐Generation Memories: First‐Principles Screening and Experimental Verification
Source: Adv Sci (Weinh). 2020 Jun 26;7(16):2001367. doi: 10.1002/advs.202001367 (PMC7435252; doi:10.1002/advs.202001367)
Supplement: Supplementary file 1 — Supporting Information [file ADVS-7-2001367-s001.pdf]

Copyright WILEY-VCH Verlag GmbH & Co. KGaA, 69469 Weinheim, Germany, 2018.

Supporting Information

Metal-Halide Perovskite Design for Next-Generation Memories: First-Principles Screening and Experimental Verification

Ju-Hyun Jung,[1,3] Seong Hun Kim,[1,3] Youngjun Park,[1,3] Donghwa Lee[1,2] and Jang-Sik Lee[1]**

[1]Department of Materials Science and Engineering, Pohang University of Science and Technology (POSTECH), Pohang 37673, Korea

[2]Division of Advanced Materials Science, Pohang University of Science and Technology (POSTECH), Pohang 37673, Korea

*Corresponding Author. E-mail: jangsik@postech.ac.kr, donghwa96@postech.ac.kr

[3] These authors contributted equally to this work.

Table S1. Data for calculating Pearson-correlation coefficients. Set voltage, defect formation energy, and migration barrier are obtained from the reported values. The values (bandgap, Pb-X bonding length, bonding angle, tolerance factor) are obtained from first-principles DFT calculations.

	$MAPbI_3$	δ-$FAPbI_3$	$MAPbBr_3$	Orthorhombic $CsPbBr_3$
Set voltage (V)	0.7[1]	0.2[2]	1[3]	1.5[4]
Defect formation energy (eV)	0.58[5]	0.39[2]	0.925[6]	1.2[7]
Migration barrier (eV)	0.57[8]	0.48[2]	0.23[1]	0.25[9]
Bandgap (eV)	1.44	2.69	1.79	1.9
Pb-X bonding length (Å)	3.1	3.2	2.9	3.0
bonding angle (°)	166.3	70.0	167.0	152.6
Tolerance factor	0.94	1.03	0.93	0.92

Table S2. Comparison of this work with previously-reported HP-based RSM.

Device Structure	Switching voltage (V)	On/off ratio	Retention (s)	Endurance (cycles)	Ref.
Au/$CH_3NH_3PbI_3$/ITO	0.7	~10	10^4	400	[10]
Au/$CH_3NH_3PbI_{3-x}Cl_x$/FTO	0.8	< 10	10^4	100	[11]
Al/$CsPbBr_3$/PEDOT:PSS/ITO	-0.6	~ 10^2	-	50	[12]
Au/Cs4PbBr6/PEDOT:PSS/ITO	1.2	< 10^2	10^4	100	[13]
Au/$CsPb_2Br_5$/ITO	0.7	~ 10^2	10^4	500	This work

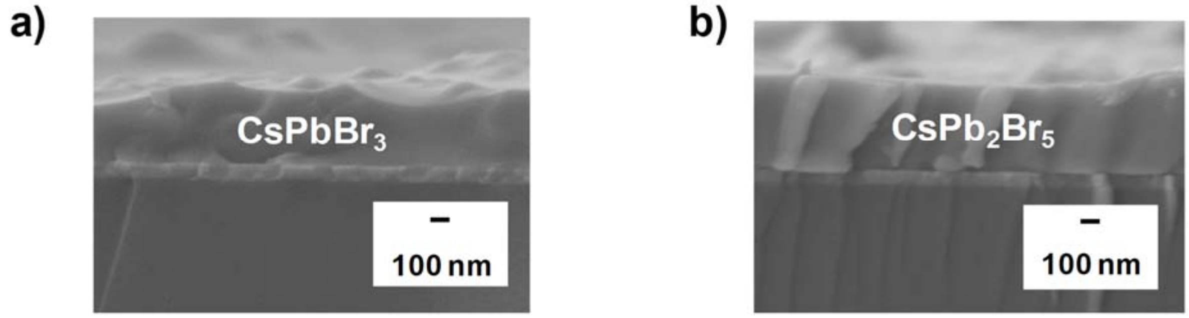

Figure S1. Cross-sectional SEM images of $CsPbBr_3$ and $CsPb_2Br_5$.

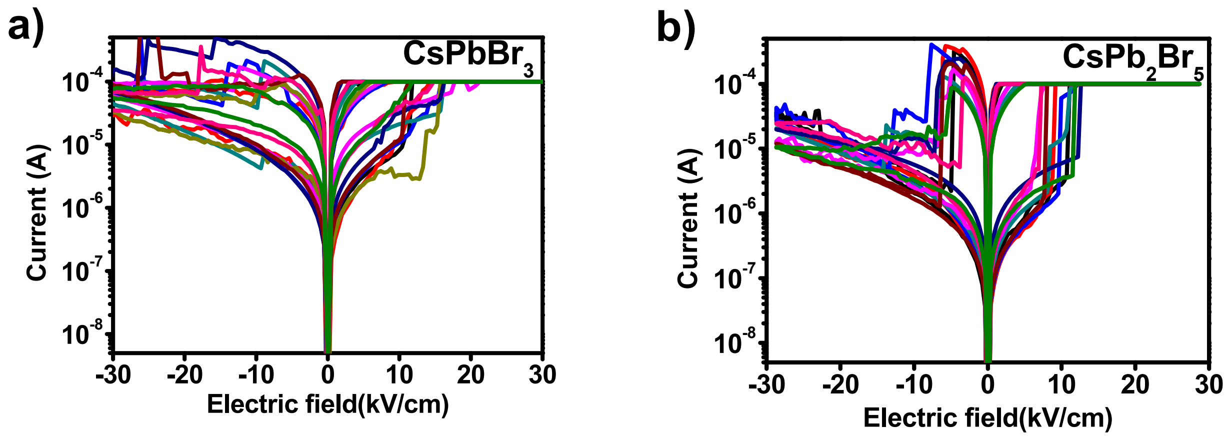

Figure S2. *I-V* curves of a) Au/$CsPbBr_3$/ITO and b) Au/$CsPb_2Br_5$ devices measured from 10 different devices.

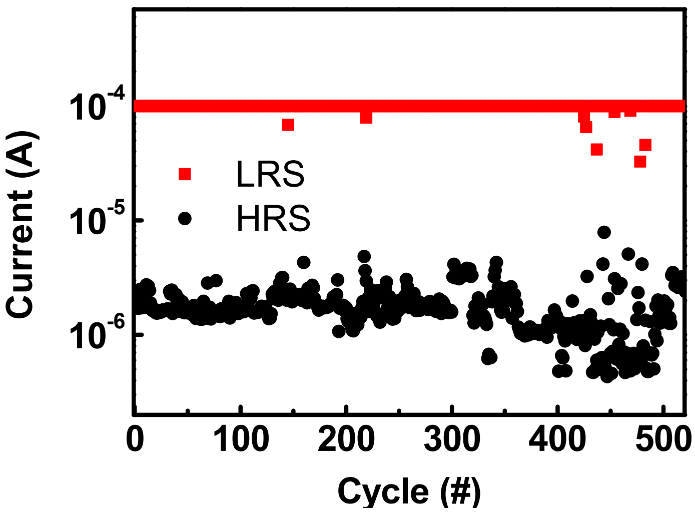

Figure S3. Endurance properties of Au/$CsPb_2Br_5$/ITO device.

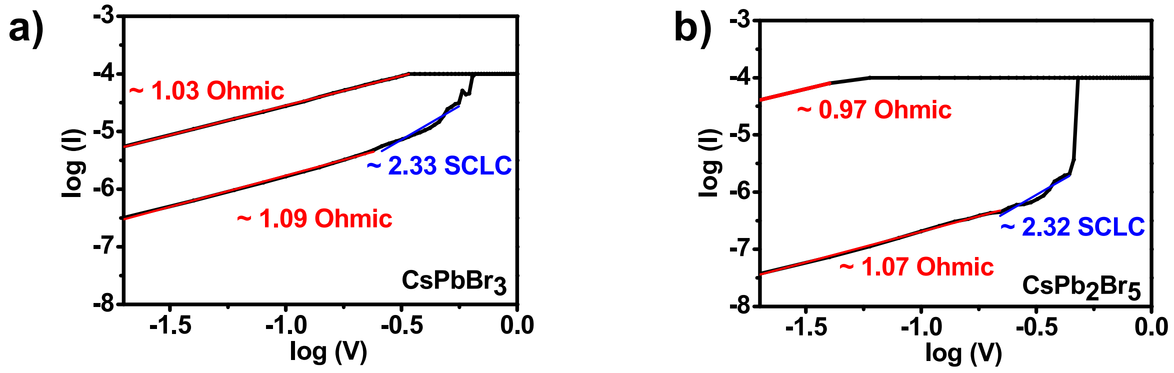

Figure S4. Log *I* - log *V* plots with fitted conduction mechanism of a) Au/$CsPbBr_3$/ITO and b) Au/$CsPb_2Br_5$/ITO.

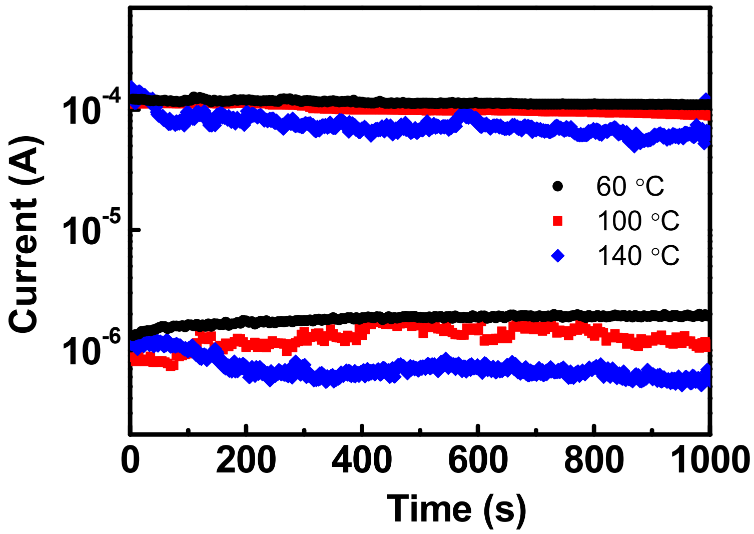

Figure S5. Data retention properties of $CsPb_2Br_5$-based RSM at 60, 100, and 140 °C.

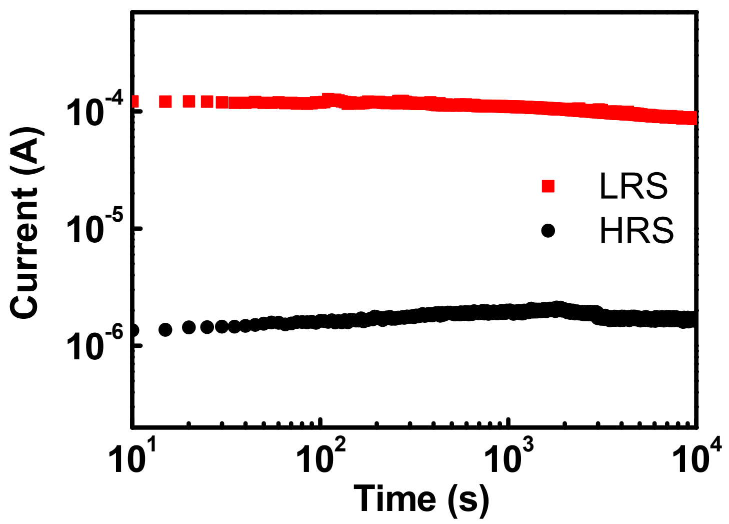

Figure S6. Data retention properties of LRS and HRS for 10^4 s at 60 °C.

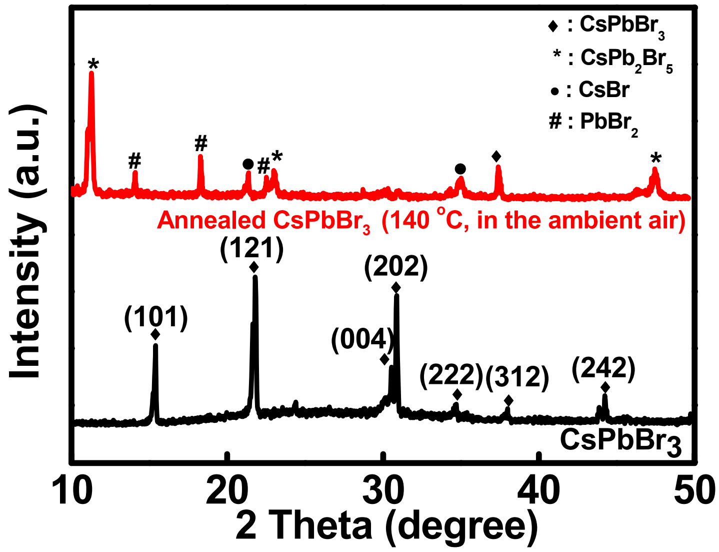

Figure S7. XRD patterns before and after annealing of $CsPbBr_3$ at 140 °C in ambient air.

Reference

[1] J. H. Heo, D. H. Shin, S. H. Moon, M. H. Lee, D. H. Kim, S. H. Oh, W. Jo, S. H. Im, *Sci. Rep.* **2017**, 7, 16586.
[2] J. M. Yang, S. G. Kim, J. Y. Seo, C. Cuhadar, D. Y. Son, D. Lee, N. G. Park, *Adv. Electron. Mater.* **2018**, 4, 1800190.
[3] X. Guan, W. Hu, M. A. Haque, N. Wei, Z. Liu, A. Chen, T. Wu, *Adv. Funct. Mater.* **2018**, 28, 1704665.
[4] H. Cai, G. Ma, Y. He, L. Lu, J. Zhang, H. Wang, *Ceram. Int.* **2019**, 45, 1150.
[5] D. Meggiolaro, F. De Angelis, *ACS Energy Lett.* **2018**, 3, 2206.
[6] A. Oranskaia, J. Yin, O. M. Bakr, J.-L. Brédas, O. F. Mohammed, *J. Phys. Chem. Lett.* **2018**, 9, 5474.
[7] J. Yin, H. Yang, K. Song, A. M. El-Zohry, Y. Han, O. M. Bakr, J.-L. Brédas, O. F. Mohammed, *J. Phys. Chem. Lett.* **2018**, 9, 5490.
[8] C. Eames, J. M. Frost, P. R. Barnes, B. C. O'regan, A. Walsh, M. S. Islam, *Nat. Commun.* **2015**, 6, 7497.
[9] J. Mizusaki, K. Arai, K. Fueki, *Solid State Ion.* **1983**, 11, 203.
[10] C. Gu, J. S. Lee, *ACS Nano* **2016**, 10, 5413.
[11] E. J. Yoo, M. Lyu, J.-H. Yun, C. J. Kang, Y. J. Choi, L. Wang, *Adv. Mater.* **2015**, 27, 6170.
[12] D. Liu, Q. Lin, Z. Zang, M. Wang, P. Wangyang, X. Tang, M. Zhou, W. Hu, *ACS Appl. Mater. Interfaces* **2017**, 9, 6171.
[13] R. Chen, J. Xu, M. Lao, Z. Liang, Y. Chen, C. Zhong, L. Huang, A. Hao, M. Ismail, *Phys. Status Solidi RRL* **2019**, 13, 1900397.
